# Recent Developments in Autism Genetic Research: A Scientometric Review from 2018 to 2022

**DOI:** 10.3390/genes13091646

**Published:** 2022-09-14

**Authors:** Mengyu Lim, Alessandro Carollo, Dagmara Dimitriou, Gianluca Esposito

**Affiliations:** 1 Psychology Program, School of Social Sciences, Nanyang Technological University, Singapore 639818, Singapore; 2 Department of Psychology and Cognitive Science, University of Trento, 38068 Rovereto, Italy; 3 Sleep Education and Research Laboratory, UCL Institute of Education, London WC1H 0AA, UK

**Keywords:** Autism Spectrum Disorder, genetics, autism, ASD, scientometrics, CiteSpace

## Abstract

Genetic research in Autism Spectrum Disorder (ASD) has progressed tremendously in recent decades. Dozens of genetic loci and hundreds of alterations in the genetic sequence, expression, epigenetic transformation, and interactions with other physiological and environmental systems have been found to increase the likelihood of developing ASD. There is therefore a need to represent this wide-ranging yet voluminous body of literature in a systematic manner so that this information can be synthesised and understood at a macro level. Therefore, this study made use of scientometric methods, particularly document co-citation analysis (DCA), to systematically review literature on ASD genetic research from 2018 to 2022. A total of 14,818 articles were extracted from Scopus and analyzed with CiteSpace. An optimized DCA analysis revealed that recent literature on ASD genetic research can be broadly organised into 12 major clusters representing various sub-topics. These clusters are briefly described in the manuscript and potential applications of this study are discussed.

## 1. Introduction

With its beginnings as a category of abnormal personality types ranging from antisocial, cyclothymic, and autistic to epileptic [1], autism became formally recognised as a disorder after Leo Kanner’s clinical description [2] and eventual inclusion in the third edition of the Diagnostic and Statistical Manual (DSM-III; [3]). Presently, Autism Spectrum Disorder (ASD) in the DSM-5 is characterised by deficits in social situations and restricted and/or repetitive behaviours, interests, or activities [4]. Recent studies estimate that ASD affects approximately 1 in 54 children in the United States (i.e., 18.52/1000), with varying estimates between 4.76 and 31.3/1000 in the European Union [5]. However, due to discrepancies in definitions, testing methods, and the age range of children tested in the national surveys, it is currently not possible to arrive at a worldwide prevalence, although most studies have concluded that prevalence rates have generally increased over time [6,7,8].

Among the scientific community, there has always been a suspicion that there was a hereditary basis to the development of autism. The first biologically grounded investigation into the etiology was conducted by MAY and Dunn [9], and made use of blood typing and antigen analysis. Less than a century later, beginning with Mendel’s discoveries in genetics [10], and in tandem with progress in biotechnological tools, genetic research in ASD is now made possible and more sophisticated than ever. Since the advent of genetic sequencing and manipulation, thousands of articles have been published on the genetic underpinnings of ASD, and many reviews synthesizing these findings have also been written (for example, see [11,12]). The latest review in this area is a meta-analysis written by Qiu et al. [13], which discussed the significant effect of polymorphisms on 12 candidate gene loci. However, rather than a singular disorder with a monogenic cause, ASD is an umbrella of neurodevelopmental abnormalities. ASD has been linked to dozens of genetic loci and hundreds of alterations from the genetic sequence to its expression, epigenetic transformation, and interactions with other physiological and environmental systems, all of which can be studied under the umbrella of ASD genetic research. Admirable attempts to summarise and report findings across these sub-categories have surfaced (e.g., Ref. [14]), but a majority of reviews in this area are restricted to only a particular aspect of ASD genetics. For example, while the narrative review by Cataldo et al. [15] focused on oxytocin and arginine-vasopressin receptors, the systematic review by Azhari et al. [16] examined the mechanisms related to the gut–brain hypothesis. Rather than narrative reviews which may organise the existing literature informally without declaring a methodology and may inevitably miss out on relevant publications [17], or meta-analyses that require a much more homogeneous set of studies and data, an alternative approach may be needed to systematically chart out the latest developments in ASD genetic research where the available literature is wide-ranging and varied.

The present paper therefore aims to make use of scientometric methods to perform a systematic review of the literature related to ASD genetic research in the last 5 years (i.e., 2018 to 2022). Further, based on the generated research clusters that arise from scientometric analysis, a brief survey of prominent citing and cited papers in each cluster will be described. Scientometry is a quantitative method of systematic review that measures the production of knowledge in a particular field by mapping out the quantitative relationships between publications [18,19]. Scientometric reviews proved useful to detect the developments of sub-specializations in topical research (e.g., [20]), where thematic research clusters are formed organically through the detection of quantitative relationships among documents. Compared to manual narrative reviews, scientometric reviews are data-driven, systematic, objective, and they are able to present findings representative of the entire scope of research with less bias [21].

## 2. Materials and Methods

### 2.1. Data Collection and Conversion

Prior to conducting scientometric analysis, relevant literature has to be systematically collated and their metadata exported. To achieve this, most scientometric reviews rely on searches on established citation databases such as Web of Science, Scopus, and other similar options. For the present review, a literature search was conducted on 24 July 2022 on Scopus with the search string: “TITLE-ABS-KEY((“ASD” OR “autism” OR autist* OR “Autism Spectrum Disorder”) AND (gene* OR geno*)) AND LIMIT-TO(LANGUAGE, “English”) AND LIMIT-TO(PUBYEAR, 2022) OR LIMIT-TO(PUBYEAR, 2021) OR LIMIT-TO(PUBYEAR, 2020) OR LIMIT-TO(PUBYEAR, 2019) OR LIMIT-TO(PUBYEAR, 2018))”. While the use of multiple databases in this step is possible, it would result in numerous duplicate entries and irregularities in citation formatting that would be difficult to resolve. Therefore, only one citation platform was chosen. Scopus was chosen over other platforms for its broader coverage in terms of number of indexed journals [22]. This method of literature search was previously adopted in [23] and is recommended by Chen [24]. Based on these criteria, a total of 14,818 articles was found to (1) be indexed with a combination of autism- and genetics-related terms in their title, abstract, or keywords, (2) be published in the English language, and (3) be published between 2018 and 2022. The research was limited to documents written in English to ensure that only the international scientific literature on the field was included in the data sample.

Unlike other systematic reviews, which rely heavily on a screening process to determine documents’ relevance, this dataset did not go through a screening process after extracting articles. This is because it is recommended that scientometric analysis on CiteSpace defers this screening process during qualitative analysis of each cluster in order to avoid systematic bias of excluding potential ambiguous terms [24]. The downloaded sample of documents was then imported into CiteSpace (version 6.1.R2) for scientometric analysis. Developed by Chen [25], CiteSpace is a freely available application built on Java that can be used to systematically map scientific literature. CiteSpace supports citation data exported from major databases such as Web of Science, PubMed and Scopus, and can be used to map temporal and structural patterns in a field of study. These patterns can be detected based on co-citations (such as in the present review), or author collaborations and directed citations. In generating these maps, clusters are produced where there are dense but common networks of co-citations (such as in the present review), representing sub-themes or specializations in a given field of research. The software also has applications in tracking the development of research over time, and is able to identify specific documents which are pivotal and have wide-ranging influence in a given field of research, as well as identify prolific authors. When importing data into CiteSpace, out of the total 948,381 references identified in the downloaded documents, 937,915 references were successfully converted, indicating a success rate of 98.90% (i.e., data loss of 1.10%). The data loss of the current work is within the acceptable bounds of 1.00% to 5.00%, and it may be considered negligible [26]. After the conversion step, the “Remove Alias” function was turned on in CiteSpace in order to eliminate repeated or identical entries.

### 2.2. Document Co-Citation Analysis

Using the converted references, a document co-citation analysis (DCA) was conducted on CiteSpace. DCA is a form of scientometric analysis that makes use of the frequency with which two or more papers are cited together (co-cited) in source articles [22,27]. The foundation of DCA rests on the assumption that higher co-citation frequencies between two or more documents are a marker of common lines of research between the cited documents [28]. Based on these principles, the final DCA network consists of two types of nodes: documents that are frequently cited together and main citing documents. To construct a DCA network, there are a variety of criteria that may be used to select the network’s nodes. Mainly, the three node selection criteria are: G-index, Top N, and Top N%. The G-index is a representation of an author’s citation score (i.e., higher G generally indicates higher author citations) and was designed as an improvement of the previously established H-index as it weighs more heavily the author’s most cited *g* publications [26,29,30,31]. To calculate G-index, the “largest number that equals the average number of citations of the most highly cited g publications” [29] is taken. The scaling factor k for the G-index criterion is introduced by CiteSpace to regulate the size of the network and can be any positive number [22]. The larger the k value, the more nodes included in the network. On the other hand, Top *N* and Top *N*% select the most cited *N* or *N*% references in a given period of time (i.e., time slice) [32]. In this review, the time slice is always kept consistent at 1 year per slice. For example, a Top *N* = 25 will select only the top 25 most cited references in each year (i.e., 2018 to 2022) for inclusion as nodes, whereas a Top *N*% = 25 will select the top quartile of most cited references in each year for inclusion as nodes.

**Figure 1:**
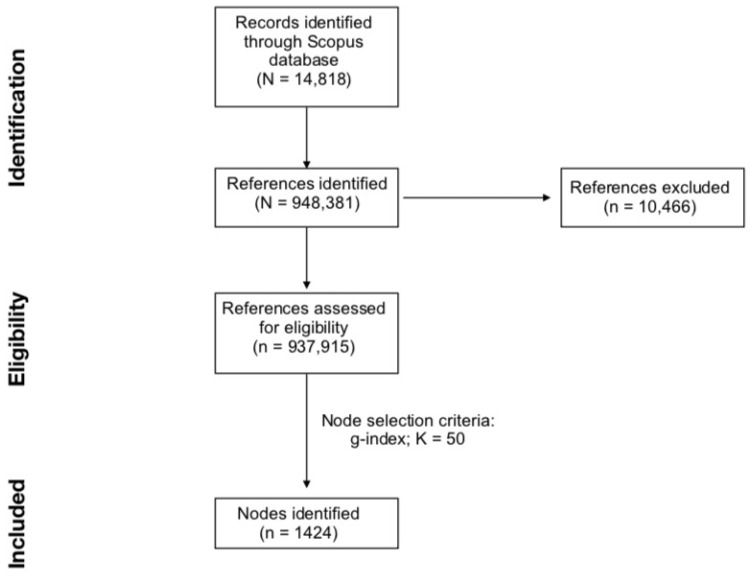
Study flow diagram (adapted from [33]).

Additionally, to allow CiteSpace to explore all possible links between older and more recent references and documents, the Look Back Years function was set to −1 (i.e., unlimited). Following the procedure of previously published papers [22], in this review, the following criteria were tested to optimize the results from the final DCA: G-index with scaling factor *k* set at 15, 25, and 50; Top *N* with *N* set at 25 and 50; Top *N*% with *N* set at 5, 10, and 15. The generated DCAs were evaluated to arrive at a final network that consists of coherent and distinct research clusters, and that is visually balanced and representative of the dataset. Particularly, the overall effects on the network’s structural metrics, the number of included nodes, and the amount of identified clusters drove the selection of the optimal node selection criteria to generate the final DCA. Based on this approach, the optimal DCA network was obtained using G-index with scaling factor *k* set at 50. Finally, clusters were obtained on CiteSpace using the “Clustering” function, which would systematically group the common co-citation pathways into clusters representing sub-themes and specializations within the research. The study flow diagram beginning with identification and retrieval from Scopus to the final number of nodes included in DCA can be found in Figure 1.

### 2.3. Metrics

Two types of metric are used to evaluate results in CiteSpace—namely, structural and temporal metrics. Structural metrics include modularity Q, silhouette score, and betweenness centrality. Modularity Q represents the degree of divisibility of a network into groups of nodes (i.e., modules or clusters) [34]. Modularity Q values range from 0 to 1, where values closer to 1 mean high network divisibility (i.e., distinct clusters and good structure) [28]. Conversely, silhouette refers to the consistency (i.e., cohesion and separation) within each cluster and ranges from −1 to 1, where values closer to 1 mean greater homogeneity within the cluster [35,36]. Finally, betweenness centrality measures the extent to which a node connects an arbitrary pair of nodes in the network [32,37]. While the values of betweenness centrality can range from 0 to 1, a value closer to 1 indicates that a publication is wide-reaching, due to its tendency to be referenced between other (less related) references and papers in the network [36]. The group of temporal metrics include citation burstness and sigma. Citation burstness represents a sudden increase in the number of citations received by a particular document over a period of time, and it allows detecting prominent publications that have received widespread attention from the scientific community [24]. Citation burstness is computed using Kleinberg’s algorithm [38]. While the lowest possible value for citation burstness is 0 (i.e., the document does not report a citation burst in its citation history), there is no theoretical upper limit for citation burstness. Finally, betweenness centrality and citation burstness values are combined with the equation (centrality + 1)${}^{burstness}$ to derive sigma. The sigma metric is an index of a document’s novelty and impact on the network.

## 3. Results

### 3.1. Structural Properties of DCA Network

**Figure 2:**
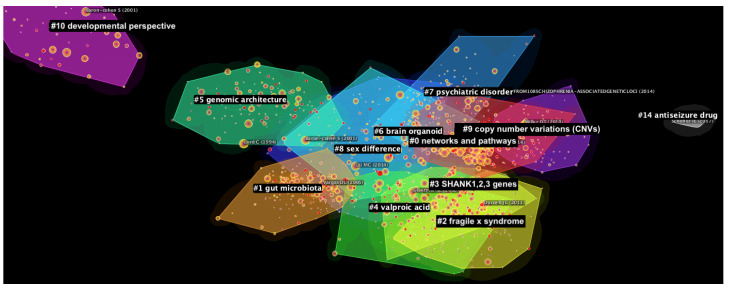
Network of publications generated through the Document Co-Citation Analysis (DCA) on the literature about the genetics of autism from 2018 to 2022. The major clusters are highlighted and divided by color.

The final DCA network consists of 1424 nodes and 7660 links (averaging approximately 5.38 links/node). Its modularity Q value is 0.6631, indicating a moderately divisible network with distinct clusters, while the average silhouette score is 0.875, indicating high homogeneity within the clusters. The network contains 12 major clusters (see Figure 2). Cluster #0 (size = 218; silhouette = 0.823; mean publication year = 2018), cluster #1 (size = 158; silhouette = 0.920; mean publication year = 2019), and cluster #2 (size = 132; silhouette = 0.850; mean publication year = 2018) are the largest clusters in the network. In terms of silhouette score, the clusters wth higher internal homogeneity were, in order, cluster #14 (silhouette = 1.000; size = 4; mean publication year = 2020), cluster #10 (silhouette = 0.985; size = 49; mean publication year = 2019), and cluster #1. The size, individual silhouette scores, average year of publication and recommended cluster labels of the major clusters are found in Table 1. Recommended labels of the clusters are generated using the log-likelihood ratio (LLR) algorithm available on CiteSpace, which identifies the most unique and yet representative terms present in each cluster [32]. While LLR provides the most accurate labels compared to other automated labelling methods on CiteSpace [39], it may nonetheless lack precision as compared to manual labelling [40]. References and papers contributing to each cluster are subsequently summarised in the Discussion section. Where renaming the clusters is deemed more appropriate, alternative cluster labels that are more representative of the references comprising the clusters are proposed.

### 3.2. Documents with a Citation Burst

A total of 163 documents are recorded with a citation burst in their history. All 163 documents have a citation burst higher than 1.9. In the network, the document with the highest citation burstness is the review of Autism Spectrum Disorder authored by Lord et al. [41] (strength of burst = 14.357; burst duration = 2). The following documents in order of citation burst strength are authored by Grove et al. [42] (strength of burst = 9.462; burst duration = 2) and Iakoucheva et al. [43] (strength of burst = 8.080; burst duration = 2). A partial list of the 20 documents with the highest citation burstness and their metrics are summarised in Table 2, in accordance with Carollo et al. [33], Gaggero et al. [39], Lim et al. [44]. It should also be noted that documents with their “Burst End” year marked as 2022 could be considered as still experiencing their citation bursts, implying that several of these references may continue to be highly cited even beyond 2022.

## 4. Discussion

To reiterate the aims of the review, the present paper will make use of scientometric methods to perform a systematic review of the literature related to ASD genetic research in the last 5 years (i.e., 2018 to 2022). Subsequently, based on the generated research clusters from scientometric analysis, a brief qualitative survey of prominent citing and cited papers in each cluster will be described.

As can be seen in Figure 2, there are some hints of overlapping contributing papers between clusters, which corroborate the quantitative finding that the generated DCA network had only moderately divisible clusters (modularity Q = 0.6631). On one hand, while it attests to the wide relevance of these papers towards sub-themes in ASD genetic research, it also shows that the different clusters are intrinsically related and rely on findings from beyond their various specializations.

In the subsequent discussion by cluster, the main citing papers that contributed to the cluster in terms of their coverage (i.e., number of references in the cluster that are cited by that paper) and the relevant cited references are reported and described, in accordance with [33]. Additionally, citing papers’ global citing score (GCS) is also reported. GCS indicates the total number of papers’ citations in Scopus. Cited references, on the other hand, are described below where applicable in terms of their frequency of being cited by the citing papers in each cluster. Where the renaming of clusters was deemed necessary, the renamed label was decided upon based on content covered by both citing documents and their corresponding references.

### 4.1. Cluster #0: Networks and Pathways

The largest cluster—namely, Cluster #0—is the least homogeneous of the major clusters, based on the silhouette metric. The major citing document in Cluster #0 is authored by Joensuu et al. [62], with a coverage of 57 documents and a GCS of 37. Based on a survey of the contributing papers, the thematic focus of the cluster appears to be on “Networks and Pathways”—the mechanisms by which genetic alterations may lead to differences in gene expression, physiological function and eventually ASD symptoms. A majority of citing documents focus on neural mechanisms and circuitry of ASD [15,63,64,65,66,67], particularly making use of evidence from chromatin remodelling and transcriptome analyses as cited papers [68,69,70]. The top 10 citing papers of Cluster #0 are seen in Table 3.

### 4.2. Cluster #1: Gut Microbiota

Cluster #1 was labelled as “Gut Microbiota”. In this cluster, the main citing document is authored by Guang et al. [71] with a coverage of 38 and a GCS of 98. As for the cluster’s label, many citing and cited papers in the cluster focus on the impact of microbes present in gut microbiomes of individuals with ASD on ASD symptoms (e.g., Refs. [45,75,76,77,78]; please see [79] for a critical analysis). A recent expansion of research in this area links gut microbiota to ASD via the gut–immune–brain axis [54,80,81], where maternal and fetal inflammatory responses to gut dysbacteriosis may have affected fetal neurodevelopment [16]. Some of the most frequently cited references in this cluster also support the gut–brain link in ASD, where gene expression, particularly those related to synaptic function and potentiation, may be altered due to gut microbiota, and that ASD severity is correlated with severity of gastrointestinal tract dysfunction [82]. Conversely, it has also been found that genetic variation may lead to differential composition of gut microbiota [83]. In this area of research, a recently published article (published a mere two days before the present review) has also reviewed the characteristics of gut microbiota that have been found to lead to ASD, as well as potential therapeutic applications to alleviate ASD symptoms by regulating the microbiome [84]. The top 10 citing papers of Cluster #1 are seen in Table 4.

### 4.3. Clusters #2 and #3: Mouse Models

Unlike the preceding clusters which had topical focuses, Clusters #2 (manually renamed “Fragile X Syndrome”) and #3 (manually renamed “SHANK1,2,3 Genes”) are discussed together primarily because of the use of animal models in their research methodology (e.g., Refs. [65,67,90,91,92,93]). The documents authored by Wang et al. [94] and by Verma et al. [91] are the major citing documents in cluster #2 with a coverage of 64 (GCS = 8) and 46 (GCS = 23), respectively. The major citing documents in Cluster #3 are authored by Wang et al. [94] and Soler et al. [95] with a coverage of 54 (GCS = 8) and 40 (GCS = 26), respectively. Mouse models in ASD research can be traced back to the work by Consorthium et al. [96], who used FMR1 knockout mice to investigate fragile X syndrome, one of the monogenic causes of ASD [97,98,99]. In fact, mouse model research in Cluster #2 focuses heavily on fragile X, where citing documents relied most frequently on Fragile X studies (e.g., Ref. [100] with a citation frequency of 52, Ref. [101] with a citation frequency of 47 and [98] with a citation frequency of 44). On the other hand, Cluster #3 uses mouse models to investigate the SHANK1, SHANK2, and SHANK3 genes that code for synaptic formation and transmissions [93,102,103,104], where mutations in these loci may also be a monogenic cause of ASD [71,91,94,105]. Therefore, proposed labels may be “Fragile X Syndrome” and “SHANK1,2,3 Genes” for Clusters #2 and #3, respectively. The top 10 citing papers of Clusters #2 and #3 are seen in Table 5 and Table 6 respectively.

### 4.4. Clusters #4 and #6: Stem Cell Technology

The major citing documents for Clusters #4 and #6 are authored by St. Clair and Johnstone [108] (coverage = 22; GCS = 13) and Lord et al. [109] (coverage = 23; GCS = 211), respectively. While Clusters #4 and #6 were originally labelled “Valproic Acid” and “Brain Organoid”, respectively, a more representative label for both clusters may be “Stem Cell Technology”, as papers in the two clusters focus on the exposure of stem cells to various molecules, including valproic acid (a molecule that is associated with higher likelihood of developing ASD [110]), to investigate the development of ASD-like phenotypes [108,111,112,113,114]. Papers in Cluster #4 use stem cells to mimic the prenatal development processes in order to trace the pathogeneses of ASD. In Cluster #6, research methodologies take it one step further through the use of brain organoids—a self-organising tissue made of these stem cells to simulate the structure and function of the human brain [115,116,117,118,119] (please see [120] for a review of organoid technology). Generally, in contrast to animal models which may be difficult to validate in human ASD [121,122], stem cell technology offers a valuable alternative in modelling genetic variants implicated in autism by using induced pluripotent stem cells (iPSC or its human-only variant, hiPSC) from the reprogramming of somatic cells. The combination of stem cell with CRISPR gene editing, three-dimensional organoid development, and in vitro to in vivo engraftment technologies opens up avenues for stem cell therapies in the future [123]. The top 10 citing papers of Cluster #4 and #6 are seen in Table 7 and Table 8.

### 4.5. Cluster #5: Genomic Architecture

In Cluster #5, the major citing document is written by Al-Dewik et al. [132] and has a coverage of 20 documents and a GCS of 5. As the term “Genomic Architectured” implies, many studies in this cluster make use of big data, such as genome-wide associations or transcriptome analyses, to uncover the genetic bases of ASD [133,134,135,136,137,138,139,140]. The top 10 citing papers of Cluster #5 are seen in Table 9.

### 4.6. Cluster #7: Psychiatric Disorder

The document by Lord et al. [41] with a coverage of 18 and a GCS of 211 is the main citing document of Cluster #7 “Psychiatric Disorder”. This cluster represents an expansion of genetic studies beyond ASD to other neurological and psychiatric disorders such as major depressive disorder [148], schizophrenia [95,108,149,150,151], obsessive-compulsive disorders [152], Parkinson’s disease [153], Tourette’s syndrome [154], as well as behavioural indicators of psychiatric disorders such as self-harm and suicidality [155], and sociability [156]. Studies in this cluster either are comparative in nature (i.e., analyzing and comparing genetic bases of the various disorders), or apply the success of genetic research in ASD to other disorders. Generally, the cluster is testament to the progress and accomplishments in the field of ASD that researchers of other psychiatric disorders may take inspiration from. The top 10 citing papers of Cluster #7 are seen in Table 10.

### 4.7. Cluster #8: Sex Difference

In Cluster #8, Rylaarsdam and Guemez-Gamboa [14] authored the major citing document with a coverage of 11 references and a GCS of 118. The current label “Sex Difference” is derived from some studies in the cluster investigating the significant discrepancy in male-to-female individuals diagnosed with ASD and the effects of sex on ASD [125,165,166,167,168], with a small extension of research into ASD and gender dysphoria [169]. Notably, one of the most frequently cited references, with a citation frequency of 112, in the cluster is a systematic review and meta-analysis by [170] on the sex ratio in ASD. The top 10 citing papers of Cluster #8 are seen in Table 11.

### 4.8. Cluster #9: Copy Number Variations (CNVs)

In Cluster #9, the major citing document is Jønch et al. [176], with a coverage of 11 and a GCS of 19. A more appropriate label for this cluster is “Copy Number Variations" (CNVs) due to the focus of contributing papers on CNV profiles in ASD. CNVs, which are the most common form of structural variations in the human genome [177] and may take the form of duplications or deletions, have been identified to be one of the risk factors for developing ASD [178]. According to the contributing papers in this cluster, the most-researched gene loci is 16p11.2 [179,180,181,182,183,184,185], which codes for proteins involved in cortical development [186]. Of the cited documents, some of the most widely cited documents indeed focused on 16p11.2 microdeletion and microduplication [187] (citation frequency of 69) and others examined CNV similarities between ASD and schizophrenia (e.g., Marshall et al. [188] with a citation frequency of 56 and Stefansson et al. [189] with a citation frequency of 50). The top 10 citing papers of Cluster #9 are seen in Table 12.

### 4.9. Cluster #10: Developmental Perspectives

Cluster #10 has Zwaigenbaum et al. [193] as major citing document with a coverage of 7 and a GCS of 20. Cluster #10 is also the most internally homogeneous cluster out of all major clusters. Contributing papers in this cluster discuss the developmental trajectory of ASD from diagnosis to adulthood [193,194,195], as well as the unique issues faced in early childhood [196,197,198] to the intersection between ASD and other health and social conditions in adulthood [199,200,201]. A major cited reference in this cluster, with a citation frequency of 100, dealt with the psychiatric co-morbidities of ASD in childhood [202]. Therefore, rather than “Autistic Adult”, which does not capture the development of ASD across a lifespan, a more appropriate label for this cluster may be “Developmental Perspectives”. The top 10 citing papers of Cluster #10 are seen in Table 13.

### 4.10. Cluster #14: Antiseizure Drug

The smallest cluster—namely, Cluster #14 “Antiseizure Drug”—has the paper authored by Hakami [208] as major citing document, with a coverage of 4 and a GCS of 2. The naming of this cluster is based on a series of two papers written by Hakami [208,209] on antiseizure drugs, and may be related to ASD due to the higher prevalence of seizure and epilepsy diagnoses among these individuals [210]. Similarly, other contributing papers focused on understanding the genetic bases of epilepsy [211,212,213] and epilepsy treatment [214,215]. In this cluster, contributing papers frequently cited references from the International League Against Epilepsy (ILAE) in their clinical descriptions and classifications of different seizures and epilepsies (e.g., Scheffer et al. [216], Fisher et al. [217,218] with citation frequencies of 43, 12, and 8, respectively). The top 10 citing papers of Cluster #14 are seen in Table 14.

### 4.11. Limitations and Future Recommendations

Despite the emergence of meaningful research clusters from scientometric analysis, this review nonetheless still has some limitations. Firstly, due to the choice of keywords used, relevant papers that had not indexed autism- and gene-related terms in their titles, abstracts, or keywords would inevitably be excluded from the dataset [25]. Papers that instead discussed ASD under the larger umbrella of neurodevelopmental disorders may also be missed as a result. Of course, unpublished but relevant papers would also be excluded from the analysis [222].

Secondly, DCA relies on the theory that higher co-citation frequencies between two or more documents are a marker of common lines of research or of emerging research trends within the field of study [28]. However, there are some caveats to a simplistic interpretation of DCA analyses. For example, a purely quantitative measure of co-citation does not reveal the relationship between two papers. A citing paper may be in agreement or disagreement with the cited reference [33]. Therefore, it is recommended by scientometricians that analysis be accompanied with a qualitative discussion [223] in order to fully understand the context of the clusters. Furthermore, as can be seen in the mean years of publication in the above clusters, DCA tends to under-represent newer publications as compared to older publications, as later publications tend to have fewer citations. Thus, it is important to consider here that citation numbers are not wholly indicative of the paper’s importance or impact.

Finally, it must be emphasised that findings regarding the citation burstness and duration are not conclusive, due to the short time period we used to conduct the scientometric review. While the search results yielded a significant amount of literature based on the time period under consideration (approximately 14 thousand entries), an alternative search without publication year limitations actually yielded 37 thousand entries, and would be more appropriate in assessing citation bursts of documents over time. Nonetheless, it was the intention of the present paper to conduct a review of only the most recent studies in the field of ASD and genetics, particularly because of the fast-paced nature of technological advancements. Future studies that are more interested in the development of this research over time may instead opt for a full coverage of the literature from its inception to the present day.

## 5. Conclusions

It is hoped that findings from this scientometric review will encourage researchers to make full use of the available literature in this field and integrate findings from various clusters and subspecialties into their future work. Additionally, it is noted that none of the clusters predominantly focused on the translation of laboratory findings to clinical applications, as well as the development of interdisciplinary treatments with medical, psychological, and occupational perspectives. The future of genetic research in ASD may see more interdisciplinary collaborations to facilitate the process from ‘bench to bedside’. Nonetheless, genetic findings on their own have exciting potential in personalized medicine [14], as well as genetic counselling and early intervention strategies [224].

## Figures and Tables

**Table 1:** Details of the 12 major clusters identified with the document co-citation analysis (DCA). Log-Likelihood Ratio (LLR) label is automatically generated by the software.

Cluster ID	Size	Silhouette	Mean Publication Year	LLR Label	Suggested Label
0	218	0.823	2018	Intellectual Disability	Networks and Pathways
1	158	0.920	2019	Gut Microbiota	Gut Microbiota
2	132	0.850	2018	Mouse Model	Fragile X Syndrome
3	120	0.885	2018	Mutant Mice	SHANK1,2,3 Genes
4	119	0.831	2018	Valproic Acid	Valproic Acid
5	110	0.919	2019	Genomic Architecture	Genomic Architecture
6	106	0.825	2019	Brain Organoid	Brain Organoid
7	102	0.893	2020	Psychiatric Disorder	Psychiatric Disorder
8	72	0.905	2019	Sex Difference	Sex Difference
9	59	0.911	2018	Autism Spectrum Disorder	Copy Number Variations (CNVs)
10	49	0.985	2019	Autistic Adult	Developmental Perspectives
14	4	1.000	2020	Antiseizure Drug	Antiseizure Drug

**Table 2:** Identifying characteristics of 20 documents with higher citation burtness metric generated in the document co-citation analysis (DCA).

Reference	Citation Burstness	Publication Year	Burst Begin	Burst End	Duration	Betweenness Centrality	Sigma
Lord et al. [41]	14.357	2018	2020	2022	2	0.0010	1.01
Grove et al. [42]	9.462	2019	2020	2022	2	0.0128	1.13
Iakoucheva et al. [43]	8.080	2019	2020	2022	2	0.0001	1.00
Sharon et al. [45]	7.827	2019	2020	2022	2	0.0066	1.05
Ruzzo et al. [46]	7.389	2019	2020	2022	2	0.0100	1.08
Kim et al. [47]	7.172	2011	2018	2019	1	0.0000	1.00
Abraham et al. [48]	6.816	2017	2020	2022	2	0.0031	1.02
Lim et al. [49]	6.702	2017	2019	2020	1	0.0003	1.00
Yang et al. [50]	6.693	2012	2018	2019	1	0.0013	1.01
Lee et al. [51]	6.311	2019	2020	2022	2	0.0006	1.00
Nowakowski et al. [52]	6.311	2017	2020	2022	2	0.0068	1.04
Velmeshev et al. [53]	6.311	2019	2020	2022	2	0.0013	1.01
Matta et al. [54]	6.311	2019	2020	2022	2	0.0011	1.01
Estes and McAllister [55]	6.214	2015	2018	2019	1	0.0020	1.01
Goines and Ashwood [56]	6.058	2013	2020	2022	2	0.0011	1.01
Pantelis et al. [57]	6.058	2014	2020	2022	2	0.0004	1.00
Yuen et al. [58]	5.975	2015	2018	2019	1	0.0008	1.01
Antoine et al. [59]	5.909	2019	2020	2022	2	0.0074	1.04
Schafer et al. [60]	5.805	2019	2020	2022	2	0.0031	1.02
Stahl et al. [61]	5.805	2019	2020	2022	2	0.0019	1.01

**Table 3:** Top 10 citing papers in Cluster #0 identified using DCA.

Title	Coverage	Global Citing Score
Joensuu et al. [62]	57	37
Gandhi and Lee [67]	52	9
Guang et al. [71]	47	98
Garcia-Forn et al. [72]	45	8
Hui et al. [65]	44	8
Diaz-Caneja et al. [73]	44	11
Alonso-Gonzalez et al. [63]	40	25
DiCarlo and Wallace [74]	40	2
Eyring and Geschwind [66]	39	5
Iakoucheva et al. [43]	39	94

**Table 4:** Top 10 citing papers in Cluster #1 identified using DCA.

Title	Coverage	Global Citing Score
Guang et al. [71]	38	98
Patel et al. [85]	37	9
Yang and Shcheglovitov [86]	33	10
Panisi et al. [87]	30	21
Matta et al. [54]	27	78
Zheng et al. [75]	26	8
DiCarlo and Wallace [74]	25	2
Liu et al. [88]	25	11
Lombardo et al. [89]	25	73
Fattorusso et al. [77]	25	140

**Table 5:** Top 10 citing papers in Cluster #2 identified using DCA.

Title	Coverage	Global Citing Score
Wang et al. [94]	64	8
Verma et al. [91]	46	23
Gandhi and Lee [67]	43	9
Joensuu et al. [62]	40	37
Guang et al. [71]	34	98
Sungur et al. [93]	34	12
Bagni and Zukin [97]	33	109
Chaudry and Vasudevan [106]	31	0
Patel et al. [85]	31	9
Möhrle et al. [90]	28	21

**Table 6:** Top 10 citing papers in Cluster #3 identified using DCA.

Title	Coverage	Global Citing Score
Wang et al. [94]	54	8
Soler et al. [95]	40	26
Mossa et al. [103]	35	19
Yoo et al. [104]	35	22
Ali Rodriguez et al. [107]	34	10
Joensuu et al. [62]	33	37
Sungur et al. [93]	31	12
Yoo et al. [102]	29	15
Yang and Shcheglovitov [86]	29	10
Verma et al. [91]	29	23

**Table 7:** Top 10 citing papers in Cluster #4 identified using DCA.

Title	Coverage	Global Citing Score
St. Clair and Johnstone [108]	22	13
Tartaglione et al. [111]	19	28
Hui et al. [65]	18	8
Filice et al. [124]	17	18
Rylaarsdam and Guemez-Gamboa [14]	16	118
Napolitano et al. [125]	16	0
DiCarlo and Wallace [74]	14	2
Fink and Levine [112]	14	14
Patel et al. [85]	14	9
Nakai et al. [92]	14	24

**Table 8:** Top 10 citing papers in Cluster #6 identified using DCA.

Title	Coverage	Global Citing Score
Lord et al. [109]	23	211
Courchesne et al. [126]	15	40
Hoffmann et al. [127]	15	14
Ilieva et al. [115]	14	39
Hong et al. [128]	12	31
Chan et al. [116]	12	12
Niu and Parent [129]	12	18
Fetit et al. [130]	11	6
Griesi-Oliveira et al. [131]	10	22
Hui et al. [65]	10	8

**Table 9:** Top 10 citing papers in Cluster #5 identified using DCA.

Title	Coverage	Global Citing Score
Al-Dewik et al. [132]	20	5
Culotta and Penzes [141]	18	12
Breen et al. [133]	14	13
Gordon and Geschwind [142]	13	7
Prem et al. [143]	13	7
Muhle et al. [144]	12	76
Grabrucker [145]	14	2
Saxena et al. [146]	12	5
Scuderi and Verkhratsky [147]	11	8
Fink and Levine [112]	11	14

**Table 10:** Top 10 citing papers in Cluster #7 identified using DCA.

Title	Coverage	Global Citing Score
Lord et al. [41]	18	211
Park et al. [157]	16	23
Jiang et al. [158]	15	0
Urresti et al. [159]	12	11
Walker et al. [160]	12	62
Willsey et al. [161]	12	1
Hoffmann et al. [127]	12	14
Sullivan and Geschwind [162]	12	156
Rees and Owen [163]	12	28
Mullins et al. [164]	11	94

**Table 11:** Top 10 citing papers in Cluster #8 identified using DCA.

Title	Coverage	Global Citing Score
Rylaarsdam and Guemez-Gamboa [14]	11	118
Lord et al. [41]	10	211
Napolitano et al. [125]	9	0
Rujeedawa and Zaman [165]	9	0
Lai et al. [171]	8	53
Kallitsounaki and Williams [169]	6	0
Müller and Fishman [172]	6	47
Wilson et al. [173]	6	7
Howes et al. [174]	6	105
Yuen et al. [175]	6	12

**Table 12:** Top 10 citing papers in Cluster #9 identified using DCA.

Title	Coverage	Global Citing Score
Jønch et al. [176]	11	19
Egolf et al. [179]	11	14
Deshpande and Weiss [190]	11	22
Lengyel et al. [180]	10	1
Takumi and Tamada [191]	9	55
Rylaarsdam and Guemez-Gamboa [14]	9	118
Kushima et al. [192]	8	114
Bristow et al. [181]	7	11
Pucilowska et al. [182]	7	39
Campbell and Granato [149]	7	3

**Table 13:** Top 10 citing papers in Cluster #10 identified using DCA.

Title	Coverage	Global Citing Score
Zwaigenbaum et al. [193]	7	20
Barros et al. [203]	6	2
Al-Dewik et al. [132]	6	5
Lacroix et al. [204]	6	1
Kirst et al. [196]	5	0
Hollin [205]	5	0
Nebel et al. [206]	5	0
Belcher et al. [199]	5	0
Rozenblatt-Perkal and Zaidman-Zait [197]	5	1
McCracken et al. [207]	5	5

**Table 14:** Top 10 citing papers in Cluster #14 identified using DCA.

Title	Coverage	Global Citing Score
Hakami [208]	4	2
Hakami [209]	4	3
Stamberger et al. [219]	3	10
Hawkins et al. [211]	3	5
Fan et al. [220]	2	4
De Maria et al. [212]	2	4
Crawford et al. [221]	2	5
Galanopoulou et al. [214]	2	6
Cali et al. [213]	2	0
Raga et al. [215]	2	6

## Data Availability

Not applicable.

## Abbreviations

The following abbreviations are used in this manuscript:

ASD	Autism Spectrum Disorder
DCA	Document Co-Citation Analysis
LLR	Log-Likelihood Ratio
GCS	Global Citing Score
ILAE	International League Against Epilepsy
